# Indocyanine green‐marked fluorescence‐guided off‐clamp versus intraoperative ultrasound‐guided on‐clamp robotic partial nephrectomy: Outcomes on surgical procedure

**DOI:** 10.1002/bco2.307

**Published:** 2023-10-31

**Authors:** Federica Mazzoleni, Davide Perri, Andrea Pacchetti, Elena Morini, Lorenzo Berti, Umberto Besana, Eliodoro Faiella, Lorenzo Moramarco, Domiziana Santucci, Davide Fior, Giorgio Bozzini

**Affiliations:** ^1^ Division of Urology Sant'Anna Hospital San Fermo della Battaglia Italy; ^2^ Division of Urology Busto Arsizio Hospital Busto Arsizio Italy; ^3^ Division of Radiology Sant'Anna Hospital San Fermo della Battaglia Italy

**Keywords:** embolization, fluorescence, indocyanine green, partial nephrectomy, robotics

## Abstract

**Objectives:**

To compare surgical and functional outcomes between off‐clamp robot‐assisted partial nephrectomy with indocyanine‐green tumour marking through preliminary superselective embolization and on‐clamp robot‐assisted partial nephrectomy with intraoperative ultrasound identification of the renal mass.

**Material and methods:**

One hundred and forty patients with a single renal mass underwent indocyanine‐green fluorescence‐guided off‐clamp robot‐assisted partial nephrectomy with preoperative superselective embolization (Group A, 70 patients) versus intraoperative ultrasound‐guided on‐clamp robot‐assisted partial nephrectomy without embolization (Group B, 70 patients). We assessed operative time, intraoperative blood loss, complications, length of stay, renal function, need for ancillary procedures and blood transfusions.

**Results:**

Mean tumour size was 24 versus 25 mm in Group A versus Group B (*p* = 0.19). Mean operative time was 86.5 versus 121.8 min (*p* = 0.02), mean blood loss was 72.8 versus 214.2 mL (*p* = 0.02), and mean haemoglobin drop on postoperative day 1 was 1.1 versus 2.6 g/dL (*p* = 0.04) in Group A versus Group B. One‐month creatinine, hospital stay and enucleated tumour volume were comparable. Ten postoperative complications occurred in Group A (13.3%) and 11 in Group B (15.3%). Following superselective embolization, no patients required blood transfusions versus two patients in Group B. Postoperative selective renal embolization was needed in one case per group.

**Conclusions:**

Preoperative superselective embolization of a renal mass with indocyanine‐green before off‐clamp robot‐assisted partial nephrectomy significantly reduces operative time and intraoperative blood loss compared to on‐clamp intraoperative ultrasound‐guided robot‐assisted partial nephrectomy. A longer follow‐up is needed to establish the effect on renal function.

## 1 INTRODUCTION

In the last decades, the increasing surgical experience and the centralization of cases to referral centres, together with the advantages provided by better imaging techniques and robot‐assisted surgery, have pushed the boundaries of nephron‐sparing surgery to bigger and complex renal masses.^1^, ^2^, ^3^, ^4^ However, partial nephrectomy (PN) may be challenging even when managing some exophytic lesions. Identifying tumour edges could be difficult if a thick, tight layer of perirenal fat is present. In these situations, perioperative outcomes might be worse, including longer operative time and greater estimated blood loss.^5^, ^6^

Renal artery embolization is nowadays reserved for selected cases, such as palliation of symptoms in patients with a locally advanced renal cell carcinoma (RCC) not amenable to surgery, in the management of angiomyolipomas with acute bleeding or at high risk of spontaneous rupture. However, preliminary renal artery embolization of the renal lesion has been shown to reduce intraoperative blood loss before PN.^7^ Some controversies exist regarding the optimal time from embolization to nephrectomy. Kalman and Varenhorst stated that the optimal delay should be less than 48 h to minimize the risk of the so‐called postembolization syndrome, characterized by lumbar pain, nausea and fever.^8^ Surgery is also considered to be technically more difficult 72 h after embolization due to the formation of collateral vessels.^9^ Therefore, some authors suggest a delay of 24–48 h after embolization, whereas others suggest as minimal delay as possible.^10^, ^11^ In an Italian cohort, at an average of 6 h after superselective embolization (SSE), 50 patients underwent laparoscopic PN for a predominantly exophytic renal tumour. SSE was shown to be a valid option for laparoscopic PN, allowing to perform surgery without any clamping, reducing the estimated blood loss and the operative time.^12^ The same Italian group reported mid‐term results of SSE before laparoscopic PN stating that oncological outcome was comparable to that of open surgery and that functional results were encouraging thanks to the optimal preservation of renal function.^13^

Newer strategies have been searched with the aim of selectively marking the renal lesion. Simone et al. reported on 10 patients with endophytic masses treated with off‐clamp robot‐assisted partial nephrectomy (RAPN) following SSE with the delivery of an ICG–lipiodol mixture. Authors conclude that ICG tumour marking allows for avoiding intraoperative ultrasound (IUS), quickly identifying the renal mass and controlling tumour margins during the dissection with negligible complications.^14^ Larger series are needed to confirm the benefits of this technique. Therefore, we performed a comparison on surgical and functional outcomes between off‐clamp RAPN with ICG tumour marking through preliminary SSE and on‐clamp RAPN with IUS identification of the renal mass.

## 2 MATERIALS AND METHODS

**FIGURE 1:**
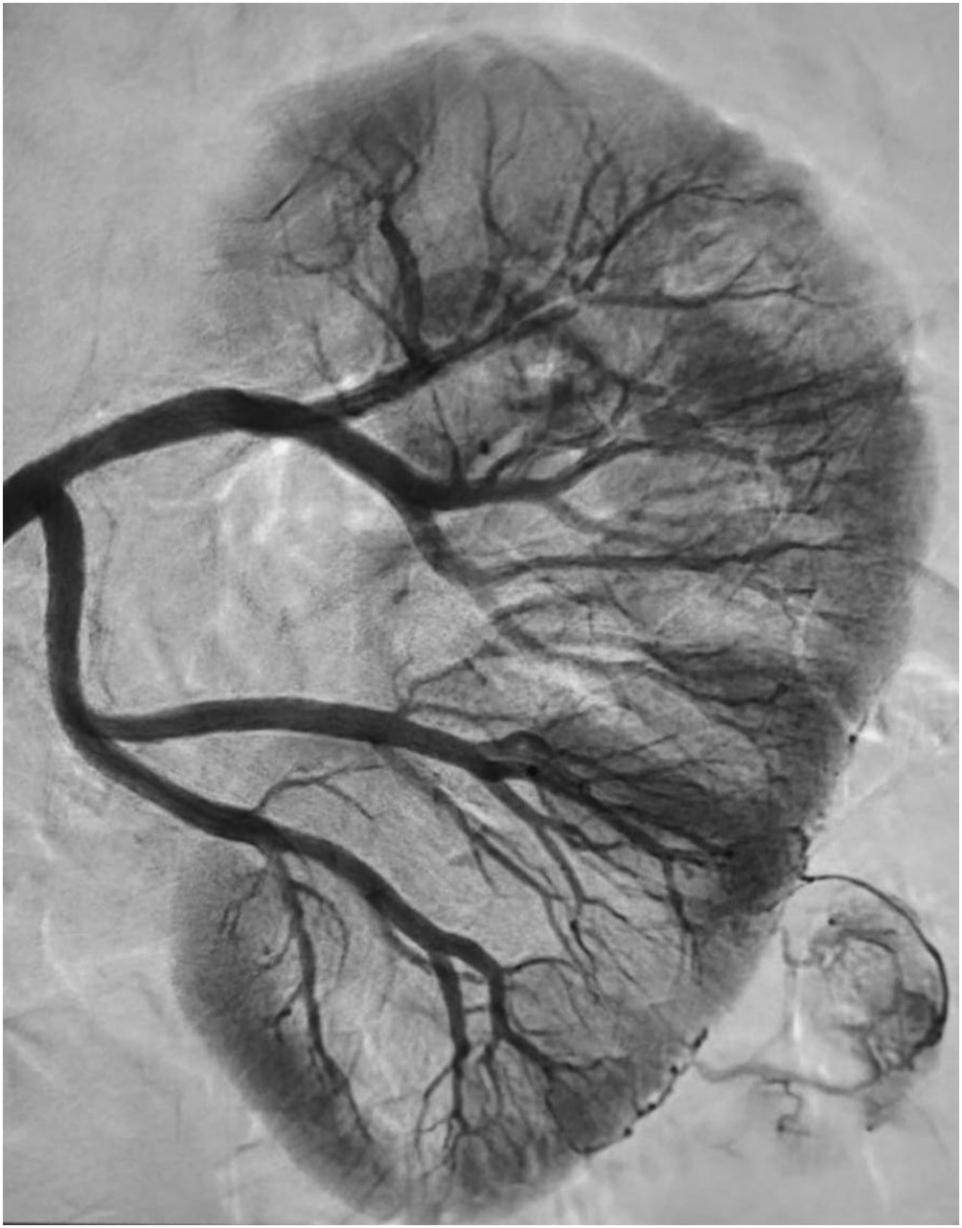
Preoperative renal angiography with visualization of the intrarenal arterial system and the renal mass vasculature.

**FIGURE 2:**
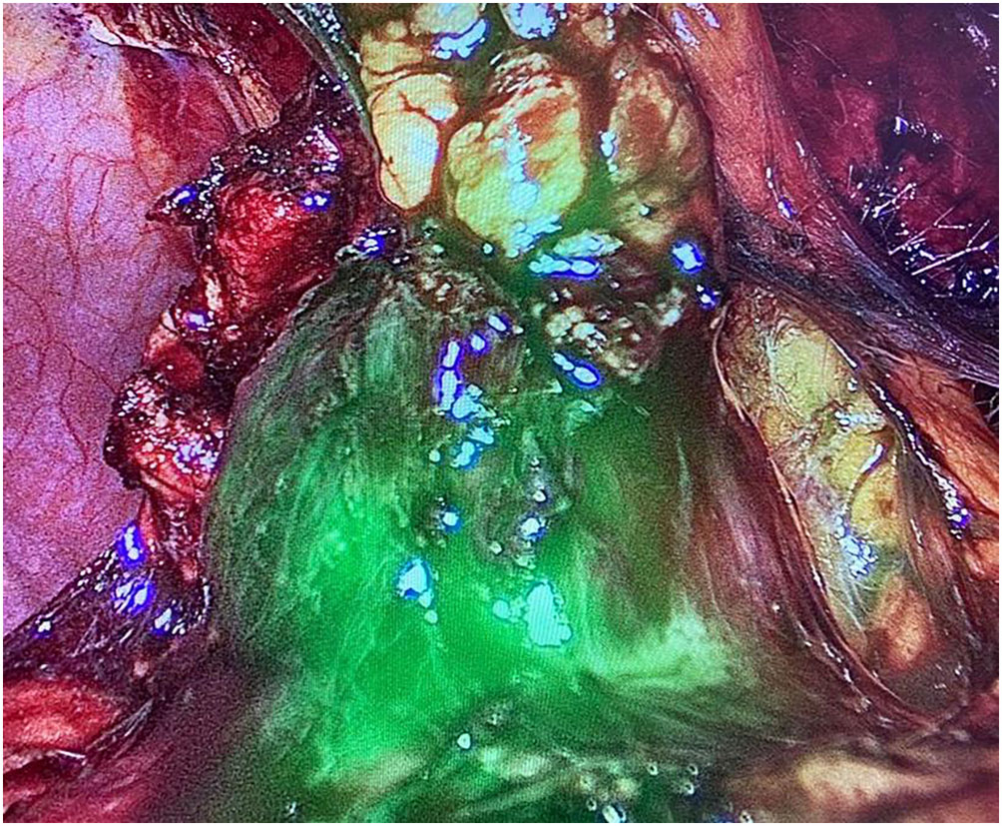
Indocyanine green‐guided localization of a renal mass during off‐clamp robot‐assisted partial nephrectomy with preoperative superselective embolization.

Between January 2019 and June 2022, 140 patients with a single renal mass suspected of malignancy were randomized to RAPN with (Group A, 70 patients) or without (Group B, 70 patients) preliminary SSE. A thoraco‐abdominal computed tomography (CT) scan with contrast enhancement was performed in all cases. Exclusion criteria were age below 18 or above 75, coagulation impairments or use of antiplatelet or anticoagulant therapy, multifocal or bilateral renal masses or cystic renal lesions, totally endophytic lesions and history of previous renal surgery. In Group A, SSE of the renal mass with ICG was performed preoperatively, and intraoperative localization of the renal mass was guided by fluorescence. Specifically, a renal angiography was performed through a transfemoral artery approach by an interventional radiologist the day before surgery in order to visualize the intrarenal arterial system and the renal mass vasculature (Figure 1). Subsequently, a superselective transarterial administration of a blend composed of indocyanine green and a non‐adhesive liquid embolic agent into tertiary or quaternary‐order arterial branches feeding the renal mass was carried out. Renal mass was localized with the assistance of intraoperative ICG fluorescence to identify tumour edges, as shown in Figure 2. Off‐clamp enucleation was then performed. Similarly to how described by Simone et al., preparation of renal hilum was carried out only in the first 10 cases. Afterwards, hilar preparation was no more performed as it was not necessary.^15^ In Group B, no embolization was performed, and IUS was used. Enucleation was performed after clamping of the main renal artery. All procedures were carried out by experienced urologists with high expertise in robotics. Similarly, embolization was performed by experienced interventional radiologists. Patients were prospectively followed and a retrospective analysis was conducted.

Surgical complexity was evaluated using the RENAL nephrometry scoring system^16^ based on imaging data. We collected preoperative data and assessed operative time, intraoperative blood loss, complication rate, length of stay, need for ancillary procedures, blood transfusions and perioperative complications. Follow‐up after surgery consisted of regular control of renal function and imaging through abdominal ultrasound and chest radiography versus thoraco‐abdominal CT scan according to the pathological stage, grade and surgical margins.

Mean and standard deviation (SD) versus numbers and proportions were used to describe continuous and categorical variables, respectively. Student's *t*‐test was used to test continuous variables conforming to a normal distribution. The chi‐square test was used for the comparison of the two study groups. Data were analysed with R software for statistical computing and graphics version 3.4.1 (R Foundation for Statistical Computing, Vienna, Austria). All statistical tests were two‐sided, with a level of significance set at *p* < 0.05. The sample size was calculated with a confidence level of 95% and a confidence interval of 5%.

## 3 RESULTS

One hundred forty patients were included. Descriptive characteristics of the cohort are summarized in Table 1. No statistically significant differences were observed according to preoperative features. Mean tumour size was 24 mm in Group A and 25 mm in Group B (*p* = 0.19), whereas the mean R.E.N.A.L. score was 8.3 in Group A versus 7.9 in Group B (*p* = 0.27).

**TABLE 1:** Descriptive characteristics of 140 patients with a single renal mass who underwent ICG fluorescence‐guided off‐clamp RAPN with preoperative SSE (Group A, 70 patients) versus IUS‐guided on‐clamp RAPN without embolization (Group B, 70 patients).

		Group A (*n* = 70)	Group B (*n* = 70)	*p*.
Age, years	Mean (SD)	63.5 (15.5)	64.1 (14.8)	0.21
Tumour size, mm	Mean (SD)	24.0 (15.0)	25.0 (17.0)	0.19
Side, *n*	R/L	33/37	34/36	0.31
Preoperative Hb (g/dL)	Mean (SD)	13.8 (2.9)	13.2 (3.1)	0.29
Preoperative Cr (mg/dL)	Mean (SD)	1.2 (0.5)	1.1 (0.4)	0.26
RENAL score	Mean (SD)	8.3 (3.4)	7.9 (4.1)	0.27
BMI (kg/m^2^)	Mean (SD)	25 (22.8–28.9)	26 (23.7–29.0)	0.43

Abbreviations: BMI, body mass index; Cr, creatinine; Hb, haemoglobin; ICG, indocyanine‐green; IUS, intraoperative ultrasound; L, left; R, right; RAPN, robot‐assisted partial nephrectomy; SD, standard deviation; SSE, superselective embolization.

Intraoperative parameters are shown in Table 2. Mean operative time was 86.5 min (SD 28.7) in Group A versus 121.8 min (SD 31.4) in Group B (*p* = 0.02), whereas mean blood loss was 72.8 ml (SD 98.4) versus 214.2 mL (SD 87.7) in Group A and Group B (*p* = 0.02), respectively. In both cases, the difference was statistically significant. No intraoperative adverse events occurred. The mean haemoglobin (Hb) drop on postoperative day 1 (POD1) was 1.1 versus 2.6 g/dL, which was statistically significant. On the contrary, postoperative creatinine at 1 month, mean hospital stay and enucleated tumour volume were not significantly different between groups (Table 2). In Group B, mean warm ischaemia time was 11 min, and overall 59 patients (84.3%) achieved Trifecta as originally described. Postoperative estimated glomerular filtration rate was not collected for all the patients, preventing the possibility to consider different definitions of Trifecta.^17^

**TABLE 2:** Perioperative outcomes of 140 patients with a single renal mass who underwent ICG fluorescence‐guided off‐clamp RAPN with preoperative SSE (Group A, 70 patients) versus IUS‐guided on‐clamp RAPN without embolization (Group B, 70 patients).

		Group A (*n* = 70)	Group B (*n* = 70)	*p*
Operative time, min	Mean (SD)	86.5 (28.7)	121.8 (31.4)	0.02
Blood loss, mL	Mean (SD)	72.8 (98.4)	214.2 (87.7)	0.02
Hb drop, g/dL	Mean (SD)	1.1 (1.4)	2.6 (3.8)	0.04
Cr at 1 month, mg/dL	Mean (SD)	1.4 (0.2)	1.6 (1.2)	0.08
Hospital stay, days	Mean (SD)	2.2 (1.9)	2.3 (1.7)	0.31
Enucleated tumour volume, g	Mean (SD)	151.1 (53.1)	148.8 (48.2)	0.22

Abbreviations: Cr, creatinine; g, grams; Hb, haemoglobin; ICG, indocyanine‐green; IUS, intraoperative ultrasound; RAPN, robot‐assisted partial nephrectomy; SD, standard deviation; SSE, superselective embolization.

Overall, we observed 10 patients with a postoperative complication in Group A (14.3%) and 11 in Group B (15.7%). The majority of complications were Clavien grade 1, specifically fever in four (5.7%) and five (7.1%) cases, paralytic ileus in three (4.3%) and two (2.9%) cases and oliguria in two (2.8%) and one (1.4%) cases in Group A and Group B, respectively. Following SSE, no patients required blood transfusions, but postoperative selective embolization was needed in one case (1.4%). In Group B, two patients (2.8%) underwent blood transfusions, and one postoperative selective embolization (1.4%) was performed. No significant difference was observed between Groups A and B for any complication or Clavien grade class, except for blood transfusions only (Table 3).

**TABLE 3:** Postoperative complications of 140 patients with a single renal mass who underwent ICG fluorescence‐guided off‐clamp RAPN with preoperative SSE (Group A, 70 patients) versus IUS‐guided on‐clamp RAPN without embolization (Group B, 70 patients).

	Group A (*n* = 70)	Group B (*n* = 70)	*p*
Overall, *n* (%)	10 (14.3)	11 (15.7)	0.16
Fever, *n* (%)	4 (5.7)	5 (7.1)	0.19
Paralytic ileus, *n* (%)	3 (4.3)	2 (2.9)	0.12
Oliguria, *n* (%)	2 (2.8)	1 (1.4)	0.40
Blood transfusion, *n* (%)	0 (0)	2 (2.8)	0.03
Embolization, *n* (%)	1 (1.4)	1 (1.4)	0.40
Clavien I, *n* (%)	9 (12.9)	8 (11.4)	0.23
Clavien II, *n* (%)	0 (0)	2 (2.9)	0.06
Clavien III, *n* (%)	1 (1.4)	1 (1.4)	0.40

Abbreviations: ICG, indocyanine‐green; IUS, intraoperative ultrasound; RAPN, robot‐assisted partial nephrectomy; SSE, superselective embolization.

Table 4 shows the results of the pathological report. The majority of renal masses were clear cell RCCs (43, 61.4% in Group A and 48, 68.6% in Group B), followed by papillary RCCs (16, 22.9% in Group A and 14, 20.0% in Group B). Papillary tumours were all type 1. Positive surgical margins were detected in two cases (2.9%) in Group A and four cases (5.7%) in Group B (*p* = 0.32) (Table 4).

**TABLE 4:** Pathological report of 140 patients with a single renal mass who underwent ICG fluorescence‐guided off‐clamp RAPN with preoperative SSE (Group A, 70 patients) vs. IUS‐guided on‐clamp RAPN without embolization (Group B, 70 patients).

	Group A (*n* = 70)	Group B (*n* = 70)	*p*
RCC clear cell, *n* (%)	43 (61.4)	48 (68.6)	0.27
RCC papillary, *n* (%)	16 (22.9)	14 (20.0)	0.21
RCC chromophobe, *n* (%)	5 (7.1)	4 (5.7)	0.15
Oncocytoma, *n* (%)	4 (5.7)	3 (4.3)	0.28
Angiomyolipoma, *n* (%)	2 (2.9)	1 (1.4)	0.28
Positive surgical margins, *n* (%)	2 (2.9)	4 (5.7)	0.32

Abbreviations: ICG, indocyanine‐green; IUS, intraoperative ultrasound; RAPN, robot‐assisted partial nephrectomy; SSE, superselective embolization.

## 4 DISCUSSION

Nephron‐sparing surgery represents the recommended treatment for organ‐confined renal masses less than 7 cm in diameter. However, evidence has demonstrated that PN is safe also when managing bigger lesions, whenever technically feasible.^18^, ^19^ Minimally invasive laparoscopic or robot‐assisted surgery is the approach of choice.^20^, ^21^, ^22^ Intra and postoperative bleeding represents the most frequent complication, which could prolong operative time and determine the need for blood transfusions or further procedures, such as renal embolization. The primary benefit of preoperative RCC embolization is a decrease in intraoperative blood loss, thus facilitating surgery and reducing operative time. However, most case series in literature include patients with advanced disease when RN is planned. Schwartz et al. reported a reduction in intraoperative transfusions and operative time but included patients with an advanced disease stage and a mean tumour size of 11.2 cm.^10^ Similarly, Luo et al. showed that preoperative renal artery embolization was significantly correlated to reduced complication rate, intraoperative blood loss and hospitalization compared to nephrectomy without embolization.^23^ Some authors have reported their experience with RCC embolization before PN with similar results. In a recent systematic review and meta‐analysis by Shanmugasundaram et al., the authors conclude that embolization of RCC prior to PN is safe and significantly reduces intraoperative blood loss.^7^ Available studies are still lacking data on the oncological outcomes, but in the experience by Simone et al., surgical margins were negative in all cases.^14^

Preoperative RCC embolization is still widely underused by urologists, and many reasons can be found. Firstly, most hospitals lack interventional radiologists able to perform SSE. Secondly, materials used for embolization have high costs, which have to be added to surgery, especially in the patients where RAPN is performed. Thirdly, favourable evidence mainly comes from case series studies. Moreover, some studies have questioned the utility of preoperative RCC embolization, with no significant difference in intraoperative blood loss and operative time compared to PN without embolization.^24^ However, embolization may become a useful tool to help the localization of the renal mass. Accordingly, our study aims to compare surgical and functional outcomes between off‐clamp RAPN with ICG tumour marking through preliminary selective RCC embolization and on‐clamp RAPN with IUS‐only identification. Some noteworthy findings need to be highlighted.

Firstly, RCC embolization and ICG‐guided localization resulted in a statistically significant reduction of 141.4 mL in mean intraoperative blood loss. Therefore, our study confirms the main benefit advocated for preliminary embolization. Whether this reduction is clinically significant is difficult to establish; however, during PN, a decrease in bleeding translates into a better visualization of the tumour bed and edges and, potentially, into a lower risk of positive surgical margins and a higher possibility of performing enucleation or resection with a thin margin of healthy parenchyma, thus preserving as much renal function as possible.

Secondly, we observed a significant reduction in operative time from a mean of 121.8 min in Group B to a mean 86.5 min in Group A. This result may be linked to the decrease mentioned above in blood loss, which allows the surgeon to perform the procedure in a more favourable condition. Surgery may be challenging and time and effort consuming. Additionally, reducing the time of procedure may also allow scheduling more surgeries in the operative programme.

Thirdly, no significant differences in postoperative outcomes according to hospitalization, need for postoperative kidney embolization and blood transfusions were observed. Therefore, preoperative SSE is a safe procedure that provides significant benefits during surgery and no significantly different consequences in postoperative management of these patients compared to those who undergo on‐clamp RAPN without embolization.

Fourthly, postoperative decrease in renal function was comparable between groups. Results show a mean increase in creatinine of 0.2 mg/dL in Group A and 0.5 mg/dL in Group B at 1 month. Therefore, preoperative SSE embolization seems not to significantly affect renal function in the very short term. However, a longer follow‐up is needed to confirm this data in the long term.

Negative implications of preoperative embolization should be considered as well. The need to hospitalize the patient the day before surgery and the materials used to perform SSE may significantly increase the costs of the whole treatment, especially in urologic departments where the surgery is carried out robotically. However, a cost‐effective analysis should take into account the benefits derived from the reduction in the operative time, with the subsequent possibility of adding more surgeries in the operative programme. This may give the possibility to perform more interventions, reduce the waiting lists and admit and discharge more patients.

Despite its strengths, the limitations of the study need to be taken into account. Firstly, the relatively low number of patients included and the short‐term follow‐up are a main concern. Secondly, none of the patients took antiplatelet or anticoagulant therapy. It would be of interest to evaluate the efficacy of preoperative SSE in patients with a higher risk of bleeding due to non‐suspendable drugs. Thirdly, all cases were performed by well‐trained surgeons with high expertise in robotic surgery. The same study could be done with surgeons at the beginning of their learning curve to eventually confirm the benefits of preliminary RCC embolization before RAPN. Lastly, the lack of a cost analysis is a limitation on the applicability of preoperative embolization with ICG before RAPN.

## 5 CONCLUSIONS

Preoperative SSE of a renal mass with ICG before off‐clamp RAPN is a safe and effective procedure that significantly reduces operative time and intraoperative blood loss compared to on‐clamp RAPN with IUS identification of the renal mass. Quick intraoperative identification of the mass with improved visualization and control of resection margins is the real advantage of this technique. Longer follow‐up is needed to establish if this procedure can affect renal function.

## AUTHOR CONTRIBUTIONS

Davide Perri, Andrea Pacchetti, Elena Morini, Lorenzo Berti and Giorgio Bozzini performed the research. Federica Mazzoleni, Davide Perri, Andrea Pacchetti, Elena Morini, Lorenzo Berti, Umberto Besana, Eliodoro Faiella, Lorenzo Moramarco, Domiziana Santucci, Davide Fior and Giorgio Bozzini designed the research study. Federica Mazzoleni, Davide Perri, Andrea Pacchetti, Elena Morini, Lorenzo Berti, Umberto Besana, Eliodoro Faiella, Lorenzo Moramarco, Domiziana Santucci, Davide Fior and Giorgio Bozzini contributed essential reagents or tools. Davide Perri, Andrea Pacchetti, Elena Morini, Lorenzo Berti and Giorgio Bozzini analysed the data. Davide Perri and Giorgio Bozzini wrote the paper.

## CONFLICT OF INTEREST STATEMENT

The authors have no competing interests.
